# Creating a honey bee consensus gene set

**DOI:** 10.1186/gb-2007-8-1-r13

**Published:** 2007-01-22

**Authors:** Christine G Elsik, Aaron J Mackey, Justin T Reese, Natalia V Milshina, David S Roos, George M Weinstock

**Affiliations:** 1 Department of Animal Science, Texas A&M University, TAMU, College Station, Texas 77843, USA; 2 Penn Genomics Institute, University of Pennsylvania, S. University Avenue, Philadelphia, Pennsylvania 19104, USA; 3 GlaxoSmithKline, S. Collegeville Road, Collegeville, Pennsylvania 19426, USA; 4 Human Genome Sequencing Center, Baylor College of Medicine, Baylor Plaza, Houston, Texas 77030, USA

## Abstract

**Background:**

We wished to produce a single reference gene set for honey bee (*Apis mellifera*). Our motivation was twofold. First, we wished to obtain an improved set of gene models with increased coverage of known genes, while maintaining gene model quality. Second, we wished to provide a single official gene list that the research community could further utilize for consistent and comparable analyses and functional annotation.

**Results:**

We created a consensus gene set for honey bee (*Apis mellifera*) using GLEAN, a new algorithm that uses latent class analysis to automatically combine disparate gene prediction evidence in the absence of known genes. The consensus gene models had increased representation of honey bee genes without sacrificing quality compared with any one of the input gene predictions. When compared with manually annotated gold standards, the consensus set of gene models was similar or superior in quality to each of the input sets.

**Conclusion:**

Most eukaryotic genome projects produce multiple gene sets because of the variety of gene prediction programs. Each of the gene prediction programs has strengths and weaknesses, and so the multiplicity of gene sets offers users a more comprehensive collection of genes to use than is available from a single program. On the other hand, the availability of multiple gene sets is also a cause for uncertainty among users as regards which set they should use. GLEAN proved to be an effective method to combine gene lists into a single reference set.

## Abstract 2

A high-quality consensus gene set for the honey bee (*Apis mellifera*) created using a new algorithm (GLEAN) is described.

## Background

Producing a gene list is one of the key deliverables in a genome project. The Honey Bee Genome Sequencing Project (HBGSP) posed several challenges in accomplishing this. At the time of this analysis, there were fewer than 100 publicly available known honey bee genes that could be used to train gene prediction algorithms. The honey bee has a large evolutionary distance from other sequenced insect genomes, and so use of orthology relationships in gene prediction programs was reduced. Moreover, some programs are more tuned to mammalian gene structures and may not perform as well with a distant genome. In addition, the honey bee has an unusually high AT content [1], which was challenging to assemble in the draft genome, resulting in some regions with less than optimal data for gene prediction. Early in the sequencing project, consortium members suspected that automated gene prediction would be challenging, because few homologs were identified in the portion of the genome with a GC content considered typical of genic regions in other metazoans. Instead, a large number of homologs aligned to AT-rich regions in honey bee. This apparent unequal distribution of genes per base composition further confounded gene discovery efforts.

Comparisons of early gene prediction results from different approaches suggested that combining sets would increase the representation of honey bee genes. Different algorithms exhibited different strengths in dealing with these issues. An additional advantage of combining sets would be that the community could work from a single official gene set. In fact, this is a general challenge for genome projects: how to choose a single gene set from multiple gene lists so that annotation and analysis can proceed from a consistent set of genes. We were then faced with this challenge of selecting gene models from individual sets to create a combined set.

GLEAN is a tool for creating consensus gene lists by integrating gene evidence. It collects evidence for genes by identifying candidate signal sites (translational start, termination, splice donor, and splice acceptor) suggested by given sources of gene evidence, and uses Latent Class Analysis to generate maximum likelihood estimates of accuracy and error rates for these signals for each gene evidence source. The posterior probability that any nucleotide site is involved in a signal is based on the evidence sources that support it and their estimated accuracy and error rates. GLEAN then uses the posterior probabilities in a dynamic programming algorithm to construct consensus gene models made up of sites that maximize the overall probability for the sites in each gene model. Some advantages of GLEAN are that it does not require a training set and that each consensus prediction is labeled with a probabilistic confidence score that reflects the underlying support for that gene model.

We used GLEAN to integrate five gene prediction sets. Our objective was to increase the number of gene models for honey bee by combining gene prediction sets, while seeking the optimal gene models when there were conflicting overlaps between sets. Here, we compare the GLEAN consensus set of gene models with the input gene prediction sets using manually annotated gene models and spliced expressed sequence tag (EST) alignments; we show that GLEAN provides an effective method to create a single reference gene set.

## Results and discussion

The overall strategy (described in Materials and methods, below) in creating an optimal honey bee gene list involved comparing a variety of gene lists with a set of manually annotated genes (which had not been included in the gene prediction evidence) and determining which gene set was superior based on two metrics. Five different sets of gene predictions were used as input to GLEAN and the output represented the sixth gene set. Two evaluations were performed. The first evaluation, to determine the utility of GLEAN, was based on a comparison with a set of 395 manually annotated gene models. These gene models were created by members of the honey bee research community using the genome assembly along with EST and cDNA sequences under study in various laboratories but not yet submitted to a public database. The EST and cDNA sequences used to construct the 395 gene models were not available to the contributors of the input gene prediction sets and were purposely omitted as evidence in generating the GLEAN consensus set. These sequences were arbitrarily selected based on availability in the community, and there were no known biases in this collection of genes. The GLEAN consensus and input gene sets were compared with these manual annotations using two metrics: the number of genes showing identical matches and the number of genes showing any match of 95% identity or greater.

Although the manually annotated gene models used in the first evaluation were high quality because of their cDNA origin, they did not allow computation of sensitivity and specificity, because they were located randomly throughout the genome. A second evaluation, using expert annotated gene models from entire scaffolds, was used to compare the sensitivity and specificity of GLEAN with those of the input gene sets. This second set of manually annotated gene models relied on protein homology and gene prediction evidence as well as cDNA evidence. Finally, the gene prediction sets were compared with spliced EST alignments to determine congruency in donor/acceptor sites.

Initial evaluations (Table 1) suggested that the GLEAN consensus set was superior to the individual gene sets. The merged GLEAN gene set had fewer gene models than most of the sets, yet it had the greatest number of perfect alignments and the highest fraction of perfectly aligned gene models. The GLEAN set had the second greatest number of genes showing any match (surpassed only by the Fgenesh set, which had three times as many gene models as GLEAN) and the greatest fraction of genes showing a match (equaling the NCBI gene list for this statistic). Thus, by these two tests the GLEAN gene set was judged to be the optimal one, with an increased number of known genes. Further evaluations described below showed that, in terms of quality, GLEAN was equal to or superior to the best gene prediction set.

### Characteristics of gene sets

General characteristics of the gene sets are shown in Table 2. GLEAN was most similar to the NCBI set in terms of gene length and transcript length. The number of single exon genes in the GLEAN set (705) was more similar to the number in the Fgenesh set (882) than to the NCBI set (194). Table 2 illustrates a challenge encountered by many gene prediction algorithms in predicting start and stop sites. GLEAN performed among the best in the proportion of complete transcripts, and only 13 of the 10,157 GLEAN gene models lacked stop codons.

### Contributions of individual sets to the consensus set

The representation of each gene set in the consensus set is shown in Table 3, using different criteria to identify overlapping gene models. The most relaxed to most stringent criteria are 80% overlap on at least one sequence, 80% overlap on both sequences, and exact match. Table 3 shows that NCBI and Fgenesh contributed to the greatest number of GLEAN gene models and exons. A more important issue might be the number of GLEAN gene models that have representation by only one set. These are the genes that would not be represented in nonconsensus sets. Table 4 shows the number of GLEAN genes models and exons represented by only one set, using the previously mentioned overlap criteria. A notable point is that a number of transcripts and exons was contributed by Fgenesh, the *ab initio *program. This illustrates a benefit of GLEAN, in that it can exploit the high sensitivity of a dataset that has low specificity.

### Evaluations

Sensitivity and specificity are shown in Tables 5 and 6 for different levels of comparison. Sensitivity and specificity were evaluated based on exact match at the gene level, transcript level, exon level, and nucleotide level. The evaluation using chromosome 15/16 manual annotations (Table 5) suggested that GLEAN was superior to all of the gene sets in all measures.

We were wary of potential observer bias because the GLEAN set was visible to the annotator when the chromosome 15/16 set was annotated. Although instructed to ignore the GLEAN models, the annotator was still able to see the GLEAN models in the chromosome 15/16 annotation, and thus might annotate genes more 'favorably' for GLEAN. To check for observer bias, the annotator created gene models on an additional scaffold without viewing the GLEAN set (Table 6). If observer bias was truly present, then we would expect GLEAN to perform poorly compared with other predictors in the scaffold evaluation.

Several of the gene sets, including GLEAN, performed poorly on the scaffold compared with the chromosome 15/16 evaluation. A possible explanation is that the performance estimates were based on a smaller number of genes on the scaffolds, and so the scaffold estimates would have greater confidence intervals (be less accurate) than the chromosome 15/16 estimates. However, what remained true is that GLEAN performed as well as or better than the other predictors in the scaffold evaluation. Furthermore, the performance not only of GLEAN but also of the other predictors decreased in the scaffold evaluation; thus, it is more likely that GLEAN's superior performance on chromosome 15/16 was not due to observer bias, as compared with an outcome in which the other predictions fare better than GLEAN in the scaffold evaluation.

Among the prediction sets, GLEAN was most congruent with aligned ESTs (Table 7). GLEAN had the greatest number of donor/acceptor splice matches to internal EST donor/acceptor sites (perfect introns), and performed among the best in the proportions of perfect donor/acceptor matches to the number of internal EST donor/acceptor sites and the total number of predicted donor/acceptor sites.

### The number of genes in honey bee

The honey bee consensus set represented a larger number of genes than were present in the NCBI set, which performed the best of all of the input sets in terms in sensitivity and specificity. However, the difference in gene number was not drastic. The consensus gene set was still heavily biased to the AT-rich regions of the honey bee genome [1]. It is reasonable to think that the combined input gene prediction programs do not represent all of the genes in the honey bee genome, and therefore the consensus set could not represent all of the genes. However, manual inspection of gene families represented in the consensus set and a tiling array experiment suggest that most genes are represented [1]. While very large genes with exons located on different scaffolds would not be predicted as complete genes, their exons would be identified as separate genes in the consensus set. Thirteen genes that crossed scaffolds were identified among 2502 manually annotated genes [2].

## Conclusion

Most eukaryotic genome projects produce multiple gene sets because of the variety of gene prediction programs, particularly those in used at NCBI and Ensembl. Because it is thought that each of the gene prediction programs currently in use has strengths and weaknesses, the multiplicity of gene sets offers users a more comprehensive collection of genes to use than is available from a single program. On the other hand, this is also a cause of uncertainty among users as to which gene set they should use. When genes are manually analyzed, a more definitive and comprehensive gene list can be provided for use by all users, for example the *Drosophila melanogaster *gene list at FlyBase [3,4].

Here we demonstrate a second method to arrive at a single gene set. The honey bee research community desired a single reference gene set so that they could proceed with functional annotation and analyses from a common list. GLEAN proved to be an effective method to combine gene lists. When compared with gold standards, the consensus set of gene models was similar or superior in quality to each of the input sets. The GLEAN consensus gene models became release 1 of the Official Honey Bee Gene Prediction set, and was the starting point for a community manual annotation effort [2]. The consensus and input gene models are available at BeeBase [5].

## Materials and methods

### Individual automated gene prediction sets

Five gene prediction sets were independently generated and are described in detail elsewhere [1]. Briefly, one set (Fgenesh) used only *ab initio *prediction, and was trained using known genes of organisms closely related to honey bee. The other sets (Ensembl, NCBI, Evolutionary Conserved Core, *Drosophila *Ortholog Set) used homology evidence, with or without an *ab initio *step. The NCBI and Ensembl pipelines relied on protein homolog and cDNA alignments. The NCBI pipeline used an *ab initio *algorithm to extend alignment-based gene predictions to start or stop codons, when necessary. The objectives of the Evolutionary Conserved Core and *Drosophila *Ortholog pipelines were different from those of the others, in that they did not attempt to predict all genes. Rather, the Evolutionary Conserved Core pipeline used alignments to proteins in UniRef to identify core orthologous groups, and the *Drosophila *Ortholog pipeline aimed to predict only one-to-one orthologs with *Drosophila melanogaster*.

### GLEAN consensus gene set

The individual gene prediction sets were integrated using GLEAN. Two additional sets of evidence, protein and EST alignments, were used in the GLEAN analysis. EXONERATE [6] was used to create alignments for metazoan SwissProt proteins, using alignments with a minimum Smith-Waterman score of 50. At locations on the assembly that had overlapping SwissProt alignment, only the greatest scoring alignment was included in the gene evidence set. EST consensus sequences were generated from 78,001 dbEST and Riken ESTs using TGICL [7]. The EST evidence set included 9,408 EST consensus sequence alignments to the genome created using EXONERATE, with a minimum 95% identity and 90% alignment coverage.

Running the GLEAN software [8] to produce the consensus gene prediction entailed three steps. First, the automated gene predictions and other evidence sources were translated into GFF2 format, and loaded into a Bio::DB::GFF-compatible MySQL relational database [9], using the bp_load_gff.pl program available within BioPerl [10]. The GLEAN program checkphase.pl was also used to ensure that all gene model CDS elements in the GFF2 files had consistently calculated intron phase values.

Second, the GLEAN program glean-lca tabulated the agreement observed for all start, stop, donor and acceptor sites in the genome predicted by any one of the individual automated gene prediction sets; from tabulations for each type of site, separate estimates of the site occurrence rate *θ*, false positive site prediction rate *α*_*i *_and false negative site predictive rate *β*_1 _for each evidence source i were obtained by maximum likelihood estimation of the following:

L∝∏x[θ∏i=1rβi1−xi(1−βi)xi+(1−θ)∏i=1rαixi(1−αi)1−xi]n(x)
 MathType@MTEF@5@5@+=feaafiart1ev1aaatCvAUfeBSjuyZL2yd9gzLbvyNv2Caerbhv2BYDwAHbqedmvETj2BSbqee0evGueE0jxyaibaiKI8=vI8tuQ8FMI8Gi=hEeeu0xXdbba9frFj0=OqFfea0dXdd9vqai=hGuQ8kuc9pgc9s8qqaq=dirpe0xb9q8qiLsFr0=vr0=vr0dc8meaabaqaciGacaGaaeqabaqadeqadaaakeaacaWGmbGaeyyhIu7aaebuaeaadaWadaqaaiabeI7aXnaarahabaGaeqOSdi2aa0baaSqaaiaadMgaaeaacaaIXaGaeyOeI0IaamiEamaaBaaameaacaWGPbaabeaaaaaaleaacaWGPbGaeyypa0JaaGymaaqaaiaadkhaa0Gaey4dIunakmaabmaabaGaaGymaiabgkHiTiabek7aInaaBaaaleaacaWGPbaabeaaaOGaayjkaiaawMcaamaaCaaaleqabaGaamiEamaaBaaameaacaWGPbaabeaaaaGccqGHRaWkdaqadaqaaiaaigdacqGHsislcqaH4oqCaiaawIcacaGLPaaadaqeWbqaaiabeg7aHnaaDaaaleaacaWGPbaabaGaamiEamaaBaaameaacaWGPbaabeaaaaaaleaacaWGPbGaeyypa0JaaGymaaqaaiaadkhaa0Gaey4dIunakmaabmaabaGaaGymaiabgkHiTiabeg7aHnaaBaaaleaacaWGPbaabeaaaOGaayjkaiaawMcaamaaCaaaleqabaGaaGymaiabgkHiTiaadIhadaWgaaadbaGaamyAaaqabaaaaaGccaGLBbGaayzxaaWaaWbaaSqabeaacaWGUbGaaiikaGqabiaa=HhacaGGPaaaaaqaaiaa=Hhaaeqaniabg+Givdaaaa@6EAB@

Where *r *represents the number of evidence sources, x is a vector of length *r *with values *x*_*i *_equal to 1 or 0, denoting whether evidence source i predicted the site to be true or not, respectively, and *n*(x) is the number of sites with equivalent evidence vectors x [11]. All observed sites were subsequently reported by glean-lca, with their corresponding estimated posterior probabilities of true gene model involvement, given the observed evidence x:

P(site=TRUE|x)=θ∏i=1rβi1−xi(1−βi)xiθ∏i=1rβi1−xi(1−βi)xi+(1−θ)∏i=1rαixi(1−αi)1−xi
 MathType@MTEF@5@5@+=feaafiart1ev1aaatCvAUfeBSjuyZL2yd9gzLbvyNv2Caerbhv2BYDwAHbqedmvETj2BSbqee0evGueE0jxyaibaiKI8=vI8tuQ8FMI8Gi=hEeeu0xXdbba9frFj0=OqFfea0dXdd9vqai=hGuQ8kuc9pgc9s8qqaq=dirpe0xb9q8qiLsFr0=vr0=vr0dc8meaabaqaciGacaGaaeqabaqadeqadaaakeaacaWGqbGaaiikaiaadohacaWGPbGaamiDaiaadwgacqGH9aqpcaWGubGaamOuaiaadwfacaWGfbGaaiiFaGqabiaa=HhacaGGPaGaeyypa0ZaaSaaaeaacqaH4oqCdaqeWbqaaiabek7aInaaDaaaleaacaWGPbaabaGaaGymaiabgkHiTiaadIhadaWgaaadbaGaamyAaaqabaaaaaWcbaGaamyAaiabg2da9iaaigdaaeaacaWGYbaaniabg+GivdGcdaqadaqaaiaaigdacqGHsislcqaHYoGydaWgaaWcbaGaamyAaaqabaaakiaawIcacaGLPaaadaahaaWcbeqaaiaadIhadaWgaaadbaGaamyAaaqabaaaaaGcbaGaeqiUde3aaebCaeaacqaHYoGydaqhaaWcbaGaamyAaaqaaiaaigdacqGHsislcaWG4bWaaSbaaWqaaiaadMgaaeqaaaaaaSqaaiaadMgacqGH9aqpcaaIXaaabaGaamOCaaqdcqGHpis1aOWaaeWaaeaacaaIXaGaeyOeI0IaeqOSdi2aaSbaaSqaaiaadMgaaeqaaaGccaGLOaGaayzkaaWaaWbaaSqabeaacaWG4bWaaSbaaWqaaiaadMgaaeqaaaaakiabgUcaRmaabmaabaGaaGymaiabgkHiTiabeI7aXbGaayjkaiaawMcaamaarahabaGaeqySde2aa0baaSqaaiaadMgaaeaacaWG4bWaaSbaaWqaaiaadMgaaeqaaaaaaSqaaiaadMgacqGH9aqpcaaIXaaabaGaamOCaaqdcqGHpis1aOWaaeWaaeaacaaIXaGaeyOeI0IaeqySde2aaSbaaSqaaiaadMgaaeqaaaGccaGLOaGaayzkaaWaaWbaaSqabeaacaaIXaGaeyOeI0IaamiEamaaBaaameaacaWGPbaabeaaaaaaaaaa@87A4@

Finally, the program glean-dp reconstructed the most likely consensus gene models from the underlying evidence, using the Viterbi dynamic programming algorithm for Hidden Markov Models (HMMs). Briefly, the consensus gene is modeled as a linearly repeating series of mutually exclusive possible states (one intergenic, three exonic, or six intronic, as described in [12]), separated by the sites identified and scored by glean-lca, which are used to provide transition probabilities between states (states have uniform emission probabilities). Thus, when the consensus gene transitions to an identical state, the posterior probability that the site is not real is included in the consensus gene path's posterior probability; otherwise, the consensus gene transitions into a new state (governed by the type of site encountered), incorporating the site's posterior probability of being true. Transitions that would introduce in-frame stop codons are disallowed, and only complete gene models are allowed (all models must begin and end with start and stop codons).

### Initial evaluation

An initial evaluation was performed to determine the utility of GLEAN before performing expert manual annotation of chromosomes. For the initial evaluation, the consensus set was compared with the input gene prediction sets as follows. A set of 395 protein sequences manually annotated using cDNA evidence by members of the honey bee research community were compared with each gene prediction set and GLEAN consensus set using FASTA [13]. The evidence used to generate these manually annotations had not been deposited to any public database and was not used in the generation of any of the input sets or the GLEAN consensus set. The two metrics used to compare the gene prediction sets with the manual models were called 'perfect alignment' and 'present'. A perfect alignment between a manually curated protein and predicted translation was counted as an alignment with 100% alignment coverage, at least 99% identity, and no gaps. A manually annotated gene was counted as present if the protein alignment was at least 95% identity, not considering gaps or alignment coverage. This stringent criterion was used to avoid counting paralogs as true matches, because we were aligning predicted translated sequences directly to manually annotated peptide sequences without knowledge of their location in the genome. The number of perfect alignments and number of present genes were weighted by number of gene models in a gene prediction set.

### Overlap, sensitivity, and specificity

Overlap of GLEAN with different gene prediction sets, sensitivity, and specificity were determined using the Eval package [14]. Overlap was computed considering three different alignment stringencies for transcripts or exons. These were as follows: 80% alignment coverage over one aligned transcript or exon (most relaxed criterion), 80% alignment coverage over both aligned transcripts or exons, and perfect alignment between transcripts or exons (most stringent criterion). In computing sensitivity and specificity, true positives were computed as perfect matches to gold standard gene models based on different levels of granularity: perfect gene matches (most stringent), perfect transcript matches, perfect exon matches, and nucleotide matches (least stringent). Gold standard sets for sensitivity and specificity were manually annotated gene models from completely annotated scaffolds.

### Creating gold standard sets

The Apollo annotation editor [15] was used to view all gene evidence sets simultaneously with protein homolog and EST alignments. An expert gene model annotator with experience in the *Drosophila *and human genome projects created gene models for entire scaffolds of honey bee chromosomes 15 and 16. The GLEAN set was visible during the chromosome 15/16 annotation, and so an additional scaffold was annotated without viewing GLEAN to check for observer bias.

### Comparison with spliced EST alignments

We determined the congruency of internal (non-UTR [untranslated region]) introns by comparing spliced EST alignments with each gene prediction set. EST contigs were aligned to the genome assembly using EXONERATE [6] with stringent criteria to ensure high quality alignments. Criteria of 99% identity, 300 nucleotide alignment length, and alignment covering 80% of the EST contig resulted in 4,490 spliced alignments with 10,837 donor/acceptor sites. EST donor/acceptor sites located between predicted start and termination codons ('internal' donor/acceptor sites) were identified for each gene prediction set. Donor and acceptor coordinates from EST alignments were compared with those of the predicted gene sets. Each donor/acceptor site was counted only once if present in multiple predicted transcripts. We determined the number of predicted donor/accepted sites that matched perfectly to internal EST donor/acceptor sites, as well as the proportions of the perfect matches to the number of internal EST donor/acceptor sites and the total number of predicted donor/acceptor sites for each gene prediction set. We also determined the numbers of matches of donors and acceptors separately.

## Additional data files

The following additional data are available with the online version of this article. Additional data file 1 contains two tables describing manually annotated and predicted gene models for the genome assembly scaffolds used in the evaluation of the consensus gene set.

## Supplementary Material

Additional File 1 Manually annotated and predicted gene models. Two tables describing manually annotated and predicted gene models for the genome assembly scaffolds used in the evaluation of the consensus gene set. Click here for file

## Figures and Tables

**Table 1:** Initial evaluation

Predicted gene set	Number of gene models	Number of perfect alignments/weighted by number of gene models	Number present/weighted by number of gene models
GLEAN	10,157	111/0.011	356/0.035
Fgenesh	32,664	100/0.003	385/0.012
NCBI	9,759	88/0.009	340/0.035
Evolutionary Conserved Core	10,966	39/0.004	284/0.026
Ensembl	27,755	32/0.0012	217/0.008
*Drosophila *Ortholog	8,878	4/0.0005	116/0.013

**Table 2:** General Statistics for GLEAN and input gene prediction sets.

		GLEAN	*Drosophila *Ortholog	Ensembl	Evolutionary Conserved Core	Fgenesh	NCBI
Genes	Count	10,157	5,842	13,397	10,960	32,576	9,414
All transcripts	Count	10,157	8,875	27,663	10,960	32,576	9,744
	Average length	8,288	4,053	5,633	6,573	2,054	9,909
	Average coding length	1,620	1,136	1,085	1,430	635	1,728
	Ave exons per	6.4	4.8	6.2	5.9	3.5	7.4
Complete transcripts	Count	9,722	460	2,923	3,918	31,003	7,966
	Average length	8,415	3,486	2,180	6,563	2,096	10,388
	Average coding length	1,644	1,112	631	1,545	631	1,808
	Ave exons per	6.5	5.2	3.7	6.3	3.5	7.8
Single exon transcripts	Count	705	34	421	275	882	194
	Average length	925	904	186	739	615	1,325
All exons	Count	64,975	27,672	13,2964	60,601	113,465	70,627
	Average length	253	239	163	243	182	234
Introns	Count	54,818	21,254	101,056	49,587	80,889	61,107
	Average length	1,235	700	1,016	1,089	571	1,287
Splice acceptors	Count	55,249	26,532	125,739	55,192	82,024	61,903
Splice donors	Count	54,831	26,444	127,760	53,653	81,469	62,762
Start codons	Count	9,726	1,639	8,110	5,501	31,441	8,949
Stop codons	Count	10,144	1,857	6,153	7,133	31,996	8,123

**Table 3:** Number (%) of GLEAN transcripts and exons with overlap to gene prediction sets

	Drosophila Ortholog	Ensembl	Evolutionary Conserved Core	Fgenesh	NCBI
Transcript 80% overlap	5,532 (55)	8,806 (84)	7,789 (81)	9,873 (98)	8,770 (93)
Transcript 80% both overlap	2,559 (256)	4,032 (40)	4,776 (47)	6,323 (62)	7,117 (70)
Transcript exact overlap	232 (2)	706 (7)	1,451 (14)	3,595 (35)	3,757 (37)
Exon 80% overlap	26,290 (41)	46,424 (72)	48,902 (75)	61,053 (94)	61,890 (95)
Exon 80% both overlap	22,566 (35)	37,805 (58)	43,023 (66)	56,442 (87)	57,128 (88)
Exon exact overlap	16,621 (26)	26,440 (41)	38,040 (59)	51,618 (79)	53,435 (82)

**Table 4:** Number (%) GLEAN transcripts and exons with overlap to only one gene prediction set

	*Drosophila *Ortholog	Ensembl	Evolutionary Conserved Core	Fgenesh	NCBI
Transcript 80% overlap	1 (0.01)	14 (0.14)	1 (0.01)	27 (0.27)	3 (0.03)
Transcript 80% both overlap	67 (0.66)	160 (1.58)	173 (1.70)	647 (6.37)	992 (9.77)
Transcript exact overlap	35 (0.34)	92 (0.91)	289 (2.85)	1431 (14.09)	1569 (15.45)
Exon 80% overlap	7 (0.01)	46 (0.07)	30 (0.05)	346 (0.53)	535 (0.82)
Exon 80% both overlap	59 (0.09)	221 (0.34)	182 (0.28)	1776 (2.73)	2224 (3.42)
Exon exact overlap	159 (0.24)	305 (0.47)	486 (0.75)	3039 (4.68)	4156 (6.40)

**Table 5:** Sensitivity and specificity using 684 manual gene models chromosomes 15 and 16

	GLEAN	*Drosophila *Ortholog	Ensembl	Evolutionary Conserved Core	Fgenesh	NCBI
Gene sensitivity	60	1	6	13	39	34
Gene specificity	65	2	5	12	15	40
Transcript sensitivity	53	1	6	12	34	30
Transcript specificity	65	1	2	12	15	41
Exon sensitivity	82	23	41	55	74	74
Exon specificity	90	56	20	61	52	77
Nucleotide sensitivity	91	37	63	72	91	87
Nucleotide specificity	97	96	79	91	82	95

**Table 6:** Sensitivity and specificity using 33 manual gene models from scaffold 1.16

	GLEAN	*Drosophila *Ortholog	Ensembl	Evolutionary Conserved Core	Fgenesh	NCBI
Gene sensitivity	39	0	0	3	36	33
Gene specificity	46	0	0	4	17	48
Transcript sensitivity	37	0	0	3	34	31
Transcript specificity	46	0	0	4	17	48
Exon sensitivity	70	26	39	54	74	72
Exon specificity	81	64	23	63	53	77
Nucleotide sensitivity	89	34	66	67	96	92
Nucleotide specificity	98	99	88	95	89	98

**Table 7:** Comparison of gene prediction sets with spliced EST alignments

	GLEAN	*Drosophila *Ortholog	Ensembl	Evolutionary Conserved Core	Fgenesh	NCBI
Unique predicted donor/acceptor sites	54,818	21,254	101,054	49,587	80,889	61,107
Internal EST donor/acceptor sites	3,255	1,467	3,157	2,504	3,227	3,233
Perfect matches to EST donor/acceptor site	2,985	1,354	2,861	2,094	2,812	2,857
Perfect matches per internal EST donor/acceptor site	0.92	0.92	0.91	0.84	0.87	0.88
Perfect matches per predicted donor/acceptor site	0.059	0.069	0.031	0.042	0.035	0.047
Donor match	3,083	1,369	2,909	2,230	3,071	3,008
Acceptor match	3,063	1,392	2,932	2,219	3,030	2,940

'Predicted donor/acceptor sites' are splice sites within predicted gene models. 'Internal EST donor/acceptor sites' are EST splice sites located between start and termination codons of predicted genes. EST, expressed sequence tag.
