# Supplementary material for: Creating a honey bee consensus gene set
Source: Genome Biol. 2007 Jan 22;8(1):R13. doi: 10.1186/gb-2007-8-1-r13 (PMC1839126; doi:10.1186/gb-2007-8-1-r13)
Supplement: Additional File 1 — Manually annotated and predicted gene models. Two tables describing manually annotated and predicted gene models for the genome assembly scaffolds used in the evaluation of the consensus gene set. [file gb-2007-8-1-r13-S1.doc]

Supplementary Table 1. Statistics for Chromosome 15/16 Gene Models

|  |  | Manual | GLEAN | Drosophila Ortholog | Ensembl | Evolutionary Conserved Core | Fgenesh | NCBI |
| --- | --- | --- | --- | --- | --- | --- | --- | --- |
| Genes | Count | 586 | 524 | 331 | 750 | 579 | 1433 | 470 |
| All Transcripts | Count | 684 | 524 | 510 | 1569 | 579 | 1433 | 484 |
|  | Average Length | 8801 | 8355 | 4261 | 6292 | 6261 | 2302 | 11155 |
|  | Average Coding Length | 1605 | 1628 | 1101 | 1128 | 1493 | 701 | 1740 |
|  | Ave Exons Per | 6.6 | 6.5 | 4.7 | 6.5 | 6.3 | 3.7 | 7.6 |
| Complete Transcripts | Count | 666 | 517 | 10 | 141 | 190 | 1425 | 459 |
|  | Average Length | 8631 | 8167 | 1323 | 2104 | 5832 | 2308 | 10915 |
|  | Average Coding Length | 1613 | 1637 | 825 | 549 | 1567 | 700 | 1746 |
|  | Ave Exons Per | 6.7 | 6.5 | 4.1 | 3.6 | 6.4 | 3.7 | 7.6 |
| Single Exon Transcripts | Count | 41 | 32 | 0 | 15 | 8 | 40 | 9 |
|  | Average Length | 938 | 1106 | 0 | 233 | 554 | 564 | 1318 |
| All Exons | Count | 3718 | 3412 | 1495 | 7568 | 3342 | 5327 | 3615 |
|  | Average Length | 249 | 250 | 243 | 160 | 239 | 189 | 230 |
| Introns | Count | 3128 | 2888 | 1126 | 5747 | 2745 | 3894 | 3142 |
|  | Average Length | 1284 | 1220 | 776 | 1198 | 958 | 588 | 1443 |
| Splice Acceptors | Count | 3117 | 2895 | 1441 | 7205 | 3069 | 3901 | 3149 |
| Splice Donors | Count | 3150 | 2888 | 1427 | 7288 | 2988 | 3895 | 3163 |
| Start Codons | Count | 684 | 517 | 73 | 393 | 279 | 1426 | 475 |
| Stop Codons | Count | 666 | 524 | 99 | 330 | 359 | 1432 | 464 |

Supplementary Table 2. Statistics For Scaffold 1.16 Gene Models

|  |  | Manual | GLEAN | Drosophila Ortholog | Ensembl | Evolutionary Conserved Core | Fgenesh | NCBI |
| --- | --- | --- | --- | --- | --- | --- | --- | --- |
| Genes | Count | 33 | 28 | 13 | 31 | 28 | 69 | 23 |
| All Transcripts | Count | 35 | 28 | 21 | 70 | 28 | 69 | 23 |
|  | Average Length | 4222 | 7581 | 4858 | 6620 | 6668 | 2182 | 7346 |
|  | Average Coding Length | 1595 | 1730 | 1388 | 1316 | 1574 | 829 | 2180 |
|  | Ave Exons Per | 5.8 | 5.8 | 62 | 5.8 | 6.3 | 3.8 | 7.7 |
| Complete Transcripts | Count | 35 | 27 | 0 | 3 | 7 | 69 | 21 |
|  | Average Length | 4222 | 7826 | 0 | 3633 | 13542 | 2182 | 7599 |
|  | Average Coding Length | 1595 | 1766 | 0 | 379 | 2164 | 829 | 2258 |
|  | Ave Exons Per | 5.8 | 5.9 | 0 | 3.0 | 8.6 | 3.8 | 7.5 |
| Single Exon Transcripts | Count | 3 | 3 | 0 | 1 | 0 | 1 | 1 |
|  | Average Length | 1144 | 1129 | 0 | 186 | 0 | 279 | 1878 |
| All Exons | Count | 188 | 162 | 77 | 315 | 160 | 260 | 176 |
|  | Average Length | 285 | 299 | 240 | 216 | 249 | 220 | 285 |
| Introns | Count | 153 | 134 | 62 | 236 | 134 | 191 | 153 |
|  | Average Length | 461 | 1222 | 762 | 1380 | 1040 | 488 | 776 |
| Splice Acceptors | Count | 154 | 135 | 73 | 302 | 147 | 191 | 153 |
| Splice Donors | Count | 154 | 134 | 75 | 301 | 142 | 191 | 155 |
| Start Codons | Count | 35 | 27 | 4 | 13 | 13 | 69 | 23 |
| Stop Codons | Count | 35 | 28 | 3 | 14 | 18 | 69 | 21 |
